# CLASP2 is involved in the EMT and early progression after transurethral resection of the bladder tumor

**DOI:** 10.1186/s12885-017-3101-3

**Published:** 2017-02-06

**Authors:** Bisong Zhu, Lin Qi, Sulai Liu, Wentao Liu, Zhenyu Ou, Minfeng Chen, Longfei Liu, Xiongbing Zu, Jun Wang, Yuan Li

**Affiliations:** 1 Department of Urology, Xiangya Hospital, Central South University, No. 87 Xiangya Road, Changsha, 410008 Hunan Province People’s Republic of China; 2 Department of Urology, The second Xiangya Hospital, Central South University, Renmin Road, Changsha, 410000 People’s Republic of China; 3 Department of Urology, The first affiliated Hospital, Zhengzhou University, No.1 Jianshe Dong Road, Zhengzhou, 450000 People’s Republic of China

**Keywords:** CLASP2 protein, Cell proliferation, Disease progression, Epithelial-mesenchymal transition, Urinary bladder neoplasms

## Abstract

**Background:**

Cytoplasmic linker-associated protein 2 (CLASP2) belongs to a family of microtubule plus-end tracking proteins that localizes to the distal ends of microtubules and regulate microtubule dynamics. We speculated that it might be involved in the epithelial-mesenchymal transition (EMT) and progression of bladder cancer (BC).

**Methods:**

Western blotting and RT-PCR were used to detect the changes at protein and mRNA levels in BC cell lines. Cell proliferation, clonogenic formation, wound healing and chamber invasion assay were used to investigate the abilities of cellular proliferation, migration and invasion. The data of BC patients treated with transurethral resection of the bladder tumor (TURBT) was collected and analyzed. The levels of mRNA of CLASP2 and EMT-related markers in tumor and urine samples were tested by RT-PCR.

**Results:**

Expressions of CLASP2 varied in four BC cell lines. Manipulation of CLASP2 expression changed EMT-related markers. CLASP2 could promote proliferation, migration and invasion in BC cell lines. The combination (CLASP2 + E-cadherin mRNA in urine) could better discriminate the patients with or without 2-years progression compared with tumor grade after TURBT.

**Conclusion:**

CLASP2 is involved in the EMT and progression of bladder urothelial cancer. Simultaneous urine-based detection of CLASP2 and E-cadherin mRNA can efficiently discriminate patients with or without 2-years progression after TURBT.

## Background

Bladder cancer (BC) is the most common carcinoma of the urinary tract. The cancer is the sixth leading cancer in men and the tenth in women throughout the world [1]. BC are classified as non-muscle-invasive bladder cancer (non-MIBC) (pTa, pT1 or carcinoma in situ [CIS]) or invasive cancer (pT2, pT3 or pT4) with the latter carrying a worse prognosis [2, 3]. Most newly diagnosed BC (75%) are non-MIBC (confined to the bladder mucosa or to the lamina propria), which are treated by transurethral resection of the bladder tumor (TURBT) followed by intravesical instillation therapy [4]. With high rates of recurrence and progression to invasive cancer, non-MIBC requires frequent follow-up and repeated treatments. Consequently, the cost per non-MIBC patient is the highest of all cancers [5]. Because of the high risk of progression, discrimination and management of non-MIBC cases with greater progressive potential to MIBC is demanding. Unfortunately, few tools with very satisfactory efficacy can be used to predict early progression in non-MIBC patients. Therefore, it is of paramount importance to better understand the molecular mechanisms involved in the initiation and progression of BC. Identification of novel biomarkers associated with disease progression and metastasis of BC and combination of their application with traditional diagnostic and prognostic parameters would contribute to development of effective strategies for the prevention, early diagnosis and treatment of BC.

Cytoplasmic linker-associated protein 2 (CLASP2) belongs to a family of microtubule plus-end tracking proteins that localizes to the distal ends of microtubules and regulates microtubule dynamics. CLASP2 functions in various microtubule-dependent processes, including cell division, cytoskeletal remodeling for cell migration, podosome regulation, and stabilization of adherens junctions [6–9]. These studies suggested CLASP2 might be implemented in cancer progression.

Epithelial-mesenchymal transition (EMT) has been reported to be involved in the critical mechanism for the acquisition of the invasive phenotype in various type of tumor, including bladder cancer [10]. EMT has been well characterized as a multistep process including dissolution of local basement membrane, loss of the epithelial polarity and tight junctions, switch of the adherens junction subtypes, and cell migration [11]. On the other hand, CLASP2 is central to coupling the organization of intracellular vesicle transport to the remodeling of cell-matrix interactions. In this process, CLASP2 promote the stability of peripheral microtubules [12]. However it is not known whether CLASP-mediated EMT and microtubule stabilization is important for cell migration.

Previous study showed that CLASP2 interacts with p120-catenin and governs microtubule dynamics at adherens junctions. The levels of expression of CLASP2 affected the localization of the other protein to cell-cell contacts and altered adherens junctions dynamics and stability [6]. Moreover, CLASP2 interacts with CLIP, binds to microtubules, and has microtubule-stabilizing effects [13–15]. Cell migration involves a preferential reduction in microtubule dynamics at the leading edge relative to the trailing edge of cells. CLASP2 could play an essential role in cytoskeletal polarization during metastasis and invasion of cancer cells [16]. Thus we speculated that CLASP2 might be involved in the EMT and progression of bladder urothelial cancer. In the present study, we first found CLASP2 could promote EMT and BC progression in vitro. Furthermore, our clinical data showed urine-based detection of CLASP2 and E-cadherin can predict early progression after TURBT.

## Methods

This study was approved by the ethical committee of Xiangya hospital. All clinical investigations had been conducted according to the principles expressed in the Declaration of Helsinki. Written consent was obtained from all the patients. Tumor of urine samples were collected as part of routine care.

### Cell lines and cell culture

The human bladder carcinoma cell lines CRL 1749, J82, T24 and HTB 9 (purchased from American Type Culture Collection, ATCC) were used in the present study. The cells were cultured in Dulbecco’s modified Eagle’s medium supplemented with 10% fetal bovine serum (FBS) in a humidified incubator with 5% CO2 at 37 °C.

### Western blot analysis

For western blot assessment, the cells were plated in culture dishes. Cells were harvested by scraping and then washed with phosphate-buffered saline (PBS). Cells were collected following centrifugation at 1,100 rpm and pellets were resuspended in lysis solution. Protein was electrophoresed using Bis-Tris gel (Invitrogen Life Technologies, Carlsbad, CA, USA). The protein was transferred to a nitrocellulose membrane (Invitrogen Life Technologies). The primary antibody was added to the culture with milk (2.5% w/v) and allowed to incubate overnight at 4 °C. The membrane was then washed prior to the addition of the appropriate horseradish peroxidase-linked secondary antibody and incubation for 1 h at room temperature. The primary antibodies were anti-CLASP2, anti-E-cadherin, anti-vimentin (Cell Signaling Technology, USA) and anti-GAPDH (Santa Cruz Biotechnology, USA). The membrane was then washed three times for 15 min each, prior to the addition of SuperSignal Chemiluminescent substrate (Pierce Biotechnology, Inc.) and then immediately visualized using a ChemiDoc Imaging system (Bio-Rad).

### Establishment of stable cell lines

The stably transduced bladder cancer cell lines aiming to either increase or inhibit the expression CLASP2 were generated. ShRNA and cDNA transduction were performed by Yinrunbio Inc, Changsha, China. Pre-mixed Lentiviral Packaging System (Biosettia, SD, USA) was utilized for viral packaging. CLASP2 expression was down-regulated by infecting cells overnight with lentiviruses expressing a CLASP2-specific shRNA (Sigma-Aldrich, USA). A non-targeting shRNA sequence was used as control. The cDNA encoding the complete coding region of CLASP2 cDNA was subcloned into the lentiviral vector. The viral titre was adjusted for optimal transduction levels in BC cells.

### Cell proliferation assay

The cell growth rates were detected using a CCK-8 cell proliferation assay (CCK-8 kit; Boster Ltd., Wuhan, China) according to our previous reports [17]. In brief, the cells (4 × 10^3^/well) were seeded in a 96-well plate and cultured at 37 °C in a 5% CO2 atmosphere. After incubation with CCK-8 solution (10 ml/well) for 1 h, the absorbance value at 450 nm was measured using a micro plate reader and analyzed at 24 h intervals, while the 650 nm served as the reference wavelength. All experiments were performed in triplicate, and the results were representative of three individual experiments.

### Clonogenic formation assays

Clonogenic survival was defined as the ability of the cells to maintain their clonogenic capacity and to form colonies. Briefly, 5000 cells were seeded into 12-well dishes in 1 mL of medium. Medium was changed every 2 days for 7–10 days to allow for colony formation. The cells were fixed with 12.5% acetic acid in 30% methanol and then stained with Brilliant Blue R. Each experiment was performed in triplicate. Finally, positive colony formations were manually counted.

### Wound healing assay

When the cells confluence reached about 70%, wounds were created by a 1000-μl pipette tip. The cells were then rinsed with medium to remove any free floating cells and debris. Medium was then added, and culture plates were incubated at 37 °C. Photographs were taken immediately and after 24 h. The area of migrating cancer cells was measured by Image J software. Duplicate wells for each condition were examined, and each experiment was repeated three times.

### Cell invasive assay

Cell invasion was determined by using a modified two chamber invasion assay with a pore size of 8 mm. For migration assay, 2 × 10^5^ HTB 9 and CRL1749 cells were seeded in serum-free medium in the upper chamber. After 12 h incubation at 37 °C, cells in the upper chamber were carefully removed with a cotton swab and the cells that had traversed the membrane were fixed in methanol and stained with leucocrystal violet. The number of invasive cells was determined by counting the leucocrystal violet stained cells. For quantification, cells were counted under a microscope in five fields (up, down, median, left, right. ×200).

### Real time semi-quantitative (RT-PCR)

Total RNA of urinary cell pellets was extracted using the RNeasy Mini Kit (Qiagen, USA). The High-Capacity cDNA archive kit (Applied Biosystems, USA) was used to synthesize complementary DNA (cDNA). The cDNA was synthesized on a PTC-200 Peltier Thermal Cycler DNA Engine (MJ Research Inc., USA). The DNA Engine Thermal Cycler with Chromo 4™ real-time detector system and Opticon Monitor software (Bio-Rad Laboratories, USA) were used for real-time PCR analysis. Cycle threshold (Ct) values were normalized to the housekeeper GAPDH gene.

The specific primers were shown as following:

- CLASP2 (forward: 5’- TTGTCGTCCTCTGTCAGTGC-3’; reverse: 5’- TGCCACGTCTTCTGTCTGTC-3’),
- E-cadherin (forward: 5’-CGGGAATGCAGTTGAGGATC-3’; reverse: 5’-AGGATGGTGTAAGCGATGGC-3’),
- Vimentin (forward: 5’-GACCTCTACGAGGAGGAGAT -3’; reverse: 5’-TTGTCAACATCCTGTCTGAA-3’)
- GAPDH (forward: 5’-ACCACAGTCCATGCCATCAC-3’; reverss: 5’-TCCACCACCCTGTTGCTGTA-3’).

### Patients and samples collection

Patients with newly diagnosed untreated bladder cancer undergoing TURBT at our institution between April 2011 and May 2013 were retrospectively selected for analysis. Radiological tests, including chest X-ray and CT, were routinely done. The surgical methods were carried out in accordance with the approved guidelines. Specimens of bladder cancer were collected at Xiangya hospital, and pathological examination of was performed by experienced genitourinary pathologists. Tumor stage, grade, size, and the number of tumors were recorded. Pathologic staging was determined according to the 2002 TNM classification, and pathologic grading was determined according to the 1973 World Health Organization classification (classified as G1, G2 and G3). Patients who lacked muscle tissue or were found with invasive tumors in TURBT specimen were excluded. Those patients who were performed with repeated TURBT 2 – 6 weeks later after the initial TURBT were not included in present study.

In all patients, cystoscopies were performed every 3 months for 2 years, then every 6 months for 5 years, and annually thereafter. Disease progression was defined as the development of Stage T1 or greater when the initial diagnosis had been Tis or Ta or the development of muscle-invasive BC (Stage T2 or greater) when the initial diagnosis was Stage T1 [18].

All the patients received either an immediate intravesical instillation (pirarubicin, 30 mg) within 24 h of TURBT or maintenance intravesical instillation for 1 year (pirarubicin, 30 mg, weekly for eight times and monthly for eight times). None of these patients was treated with BCG, because of its side effects and the government policy [19].

Total urine samples (100–150 mL) were collected before TURBT. The urine was stored at 4 °C for up to 4 h and then centrifuged. The pellet was resuspended in 1 ml of TRIzol reagent and frozen at -80 C in liquid nitrogen for further use.

### Statistical analysis

We used 2-tailed χ2 tests to determine the significance of differences between proportions. The Mann-Whitney U test or the Wilcoxon signed-rank test was used to compare continuous variables. The clinical and molecular markers likely to be associated with progression within 2 years (*P* <0.1) were selected to develop a prognostic marker panel. The predictive significance was assessed by univariable and multivariable logistic regression analysis. The candidate variables (*P* <0.1 in the univariable model) were included in the multivariable model for further analysis. The value for predicting progression was evaluated by calculating the area under the receiver operating characteristic (ROC) curve. The ROC analysis was performed using the DeLong test. [20] P values less than 0.05 were counted as significant. The statistical analysis was performed using SPSS for Windows v.13.0 and Sigmaplot for window 10.0.

## Results

### Expressions of CLASP2 varied in four BC cell lines

**Fig. 1:**
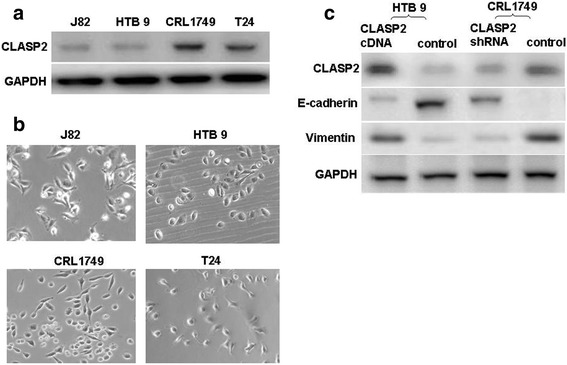
Western blotting was used to test the expression level of CLASP2 in bladder cancer cell lines including J82, HTB 9, CRL1749 and T24 (**a**). Nikon light microscope at a magnification of x200 was used to show the representative examples of the morphology of the non-manipulated cell lines (**b**). HTB 9 and CRL1749 cells were transduced with lentiviral particles expressing CLASP2 cDNA or shRNA respectively. Western blotting was used to detect the changes of expression of CLASP2 and EMT-related markers (**c**)

Four bladder cancer cell lines including J82, HTB 9, CRL1749 and T24 were tested the expression of CLASP2 at protein level. CLASP2 were stronger expressed in CRL1749 and T24 than J82 and HTB 9 cells (Fig. 1a). Figure 1b showed representative examples of the morphology of the non-manipulated four cell lines. HTB 9 and CRL1749 were selected for further experiments.

### Manipulation of CLASP2 expression changed EMT-related markers

We arbitrarily enhanced CLASP2 expression in HTB 9 cells and depleted it in CRL1749 cells by transducing lentiviral particles expressing CLASP2 cDNA or shRNA respectively. As shown in Fig. 1c the expression of the epithelial marker E-cadherin decreased significantly in HTB 9 cells following overexpression of CLASP2, whereas the expression of mesenchymal marker, vimentin was upregulated. In contrast, E-cadherin expression increased sharply in CRL1749 cells when expression of CLASP2 was inhibited (Fig. 1c). Trend with vimentin was opposite to that of E-cadherin.

### CLASP2 was involved in proliferation and clonogenic formation in BC cells

**Fig. 2:**
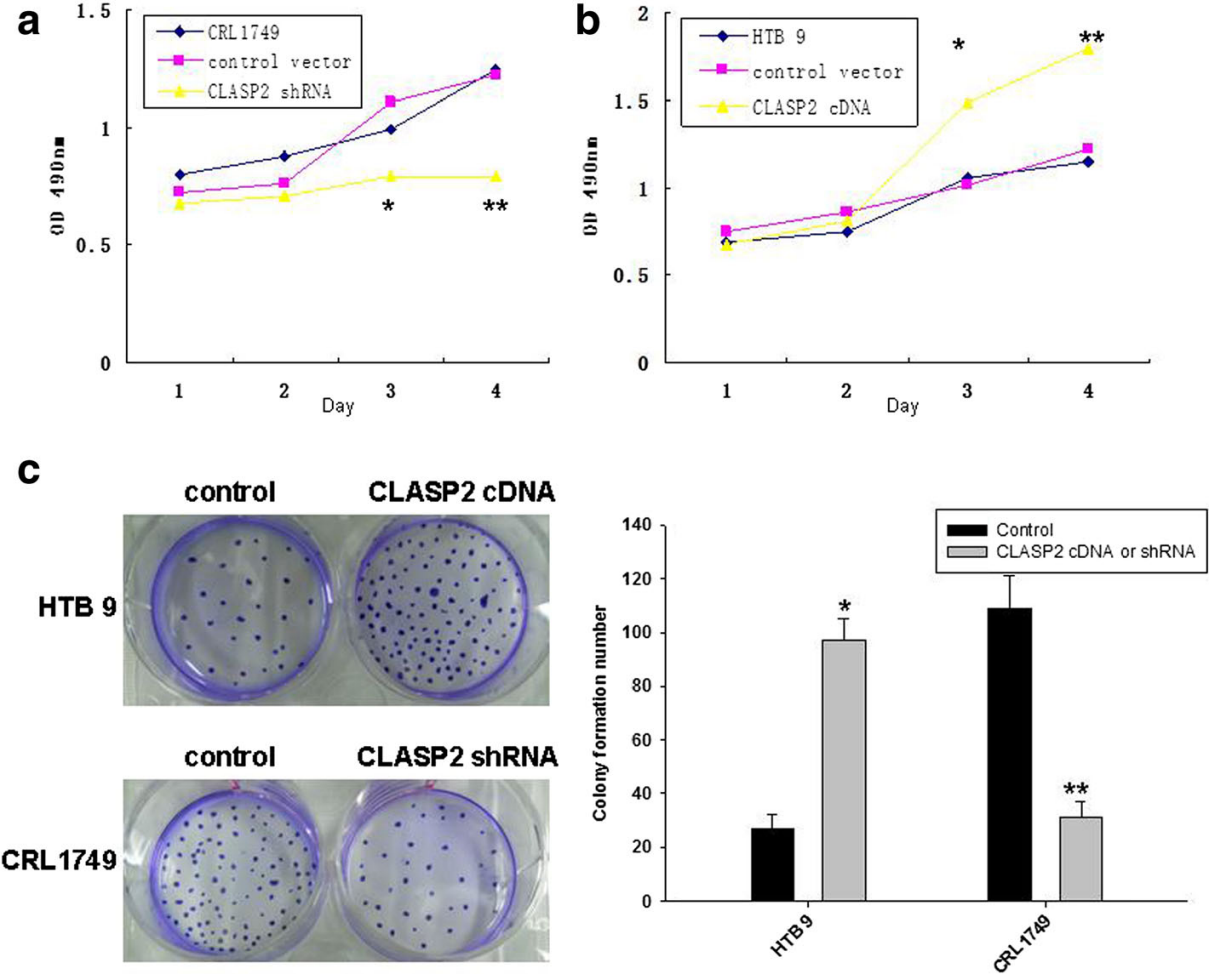
Evaluation of cell proliferation by CCK-8 assays showed that down-regulation of CLASP2 dramatically inhibited the cell proliferation rate of CRL 1749 cells (**a**), whereas overexpression of CLASP2 significantly promoted the growth of HTB9 cells (**b**). Clonogenic formation assay found that CLASP2 increased bladder cancer cells proliferation (**c**) (**P <.01, *P <0.05)

EMT was known to be related to cells proliferative and clonogenic formation ability in cancers. Several experiments were designed to investigate the association between CLASP2 and cell proliferative and clonogenic formation ability after the involvement of CLASP2 in EMT was confirmed. Cell proliferation evaluated by CCK-8 assays showed that inhibited expression of CLASP2 could decrease the cell proliferation rate of CRL 1749 cells (Fig. 2a), whereas overexpression of CLASP2 significantly promoted the growth of HTB9 cells (Fig. 2b). Clonogenic formation assay was performed to test the changes of cells proliferative ability. As the assay suggest in Fig. 2c, clonogenic formation of HTB 9 cells was promoted after transduced with CLASP2 cDNA expressing vectors, whereas impaired greatly in CRL1749 cells transduced with CLASP2 shRNA vectors.

### CLASP2 could boost migration and invasion in BC cells

**Fig. 3:**
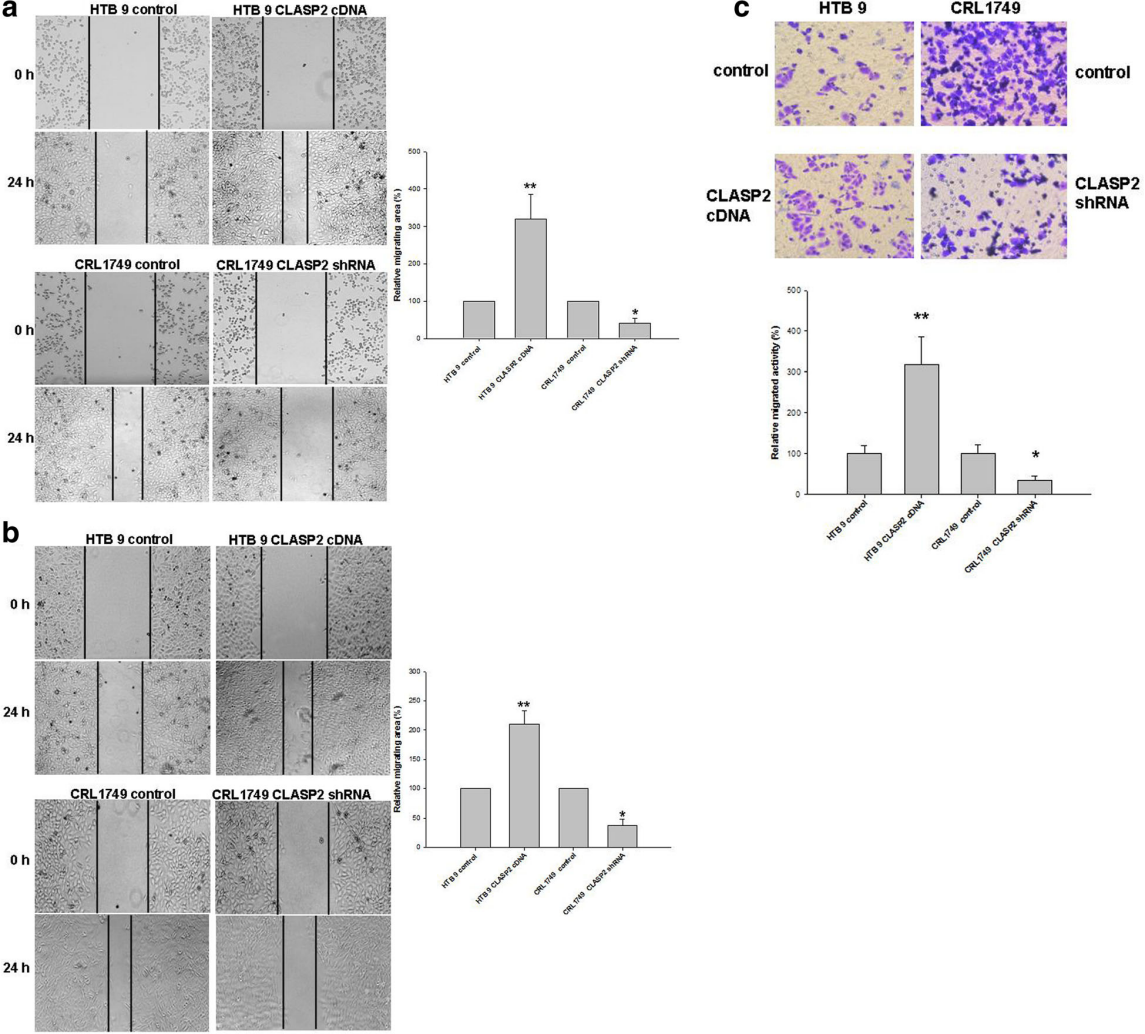
Wound healing assay suggested that CLASP2 boosted migratory abilities in BC cells both under 70% (**a**) and 100% cells confluency (**b**). Transwell invasion assay suggested that CLASP2 boosted invasive ability (**c**). The data are presented as the mean ± standard error of three independent experiments (**P <0.01, *P <0.05)

The effects of CLASP2 on cell migration and invasion were determined with wound healing and transwell invasion assay. HTB 9 cells with overexpression of CLASP2 were distinctively more migratory and invasive than negative control cells (Fig. 3a-c). Knockdown of CLASP2 by shRNA inhibited these abilities in CRL1749 cells (Fig. 3a-c). These results vividly demonstrated that CLASP2 mediated the migration and invasiveness of BC cells in vitro.

Taken together, these results suggested that overexpression of CLASP2 could facilitate the growth and aggressive phenotype of BC cells in vitro.

### Patients and baseline characteristics

Totally 102 cases with superficial bladder cancers were included. The baseline characteristics were showed in Table 1. Of the 102 patients, 61 (59.8%) had recurrence. Thirty-four (33.3%) had progression within 2 years after initial TURBT. The median time to progression was 14 months (ranging from 8 – 22 months).

**Table 1:** Clinical characteristics and expression of CLASP2 and EMT markers mRNA

Variables	Patients (*n*)	Progression within 2 years		
		No (*n*)	Yes (*n*)	*P*
Total (%)	102	68	34	
Gender				
Male	72	52	20	0.11
Female	30	16	14	
Age(years)				
< 60	38	27	11	0.61
> 60	64	41	23	
Intravesical instillation				
Immediate	29	22	7	0.31
Maintenance	73	46	27	
Tumor stage				
Ta	34	25	9	0.41
T1	68	43	25	
Tumor grade				
G1	33	26	7	0.04
G2	49	33	16	
G3	20	9	11	
Concomitant CIS				
Yes	3	1	2	0.26
No	99	67	32	
Tumor size (cm)				
< 3	59	44	15	0.07
> 3	43	24	19	
Multiplicity				
Single	44	34	10	0.06
Multiple	58	34	24	
CLASP2 mRNA (tumor)				
High	30	6	24	<0.01
Low	72	62	10	
CLASP2 mRNA (urine)				
High	55	24	31	< 0.01
Low	47	44	3	
E-cadherin mRNA (tumor)				
High	62	52	10	< 0.01
Low	40	16	24	
E-cadherin mRNA (urine)				
High	60	54	6	< 0.01
Low	42	14	28	
Vimentin mRNA (tumor)				
High	55	31	24	0.16
Low	47	37	10	
Vimentin mRNA (urine)				
High	62	36	26	0.03
Low	40	32	8	

Twenty-nine (28.4%) patients received an immediate intravesical instillation, and 73 (71.6%) patients received maintenance intravesical instillation for 1 year. The pathologic stage and grade distributions were as follows: 34 patients with Ta (33.3%) and 68 with pT1 (66.7%), and 33 (32.4%) with G1, 49 (48%) with G2 and 20 (19.6%) with G3 (19.6%), respectively. There was no significant difference between the patients with 2-years progression and those without for tumor stage (*P* = 0.41), whereas slight difference for tumor grade (*P* = 0.04). Only three patients were found with concomitant carcinoma in situ (CIS). Fifty-nine (57.8%) patients had smaller lesions (<3 cm) and 43 (42.2%) larger tumors (>3 cm), and 44 (43.1%) patients had single lesions, whereas the remaining 58 (56.9%) presented had multiple tumors (*P* <0.05). RT-PCR was used to test the mRNA levels of CLASP2 and EMT-related markers (E-cadherin and Vimentin) in bladder tumor and urines (Table 1). Significant differences were shown in the levels of CLASP2 and E-cadherin mRNA in the both tumor and urine between the patients with and without 2-years progression (*P* <0.01). However, only the mRNA level of Vimentin in the urine was found with difference (*P* = 0.03), not in the tumor (*P* = 0.16).

### Relationship between variables and progression with 2 years

Table 2 presented the results of univariable and multivariable logistic regression analysis for bladder cancer progression within 2 years. According to the optimal cut-off on the ROC curve, the levels of expression of mRNAs were categorized into low and high expression. Univariable analysis identified higher tumor grade, higher mRNA levels of CLASP2 in the tumor and urine, lower mRNA levels of E-cadherin in tumor and urine, higher level of Vimentin mRNA in the urine as risk factors for the progression within 2 years (P values seen in Table 2). Tumor size and number did not show great significance (*P* = 0.05 and *P* = 0.07), which were still included in the multivariable analysis. In multivariable analysis, tumor grade, CLASP2 and E-cadherin mRNA levels in tumor and urine were associated with increased risks of progression within 2 years (*P* values were listed in Table 2).

**Table 2:** Univariable and multivariable logistic regression analysis

	Univariable analysis		
	RR^a^	95% CI^b^	P
Gender (Male, Female)	1.12	0.91–1.22	0.67
Age(<60, >60 years)	1.03	0.98–1.17	0.61
Intravesical instillation (immediate, maintenance)	0.98	0.89–1.78	0.27
Tumor stage (Ta, T1)	1.34	1.03–1.56	0.67
Tumor grade (G1 and G2, G3)	2.78	1.05–6.93	0.01
Concomitant CIS (No, Yes)	1.52	0.21–7.43	0.66
Tumor size (<3, >3 cm)	2.11	1.29–4.32	0.05
Multiplicity (Single, Multiple)	1.98	0.98–3.92	0.07
CLASP2 mRNA (low, high in tumor)	3.83	1.12–12.22	0.02
CLASP2 mRNA (low, high in urine)	2.71	1.09–7.77	< 0.01
E-cadherin mRNA (high, low in tumor)	2.97	1.17–9.82	0.04
E-cadherin mRNA (high, low in urine)	3.24	0.97–10.81	0.02
Vimentin mRNA (low, high in tumor)	1.24	0.78–2.87	0.32
Vimentin mRNA (low, high in urine)	1.19	0.26–4.98	0.09
	Multivariable analysis^c^		
	RR	95% CI	P
Tumor stage (Ta, T1)	1.14	0.91–1.86	0.78
Tumor grade (G1 and G2, G3)	2.19	1.01–4.98	0.04
Tumor size (<3, >3 cm)	1.71	1.08–3.37	0.07
Intravesical instillation (immediate, maintenance)	1.12	0.79–1.45	0.52
Multiplicity (Single, Multiple)	2.32	1.18–4.22	0.18
CLASP2 mRNA (low, high in tumor)	2.76	1.02–10.11	< 0.01
CLASP2 mRNA (low, high in urine)	3.75	1.09–6.12	< 0.01
E-cadherin mRNA (high, low in tumor)	2.78	1.17–8.24	0.01
E-cadherin mRNA (high, low in urine)	3.34	1.12–7.45	0.01
Vimentin mRNA (low, high in tumor)	1.35	0.89–3.43	0.51
Vimentin mRNA (low, high in urine)	1.78	0.26–3.48	0.35

^a^Relative risk

^b^Confidence interval of the estimated RR

^c^Multivariable analysis adjusted for tumor grade, stage, size, multiplicity, levels of CLASP2, E-cadherin and Vimentin mRNA in the tumor or urine

**Fig. 4:**
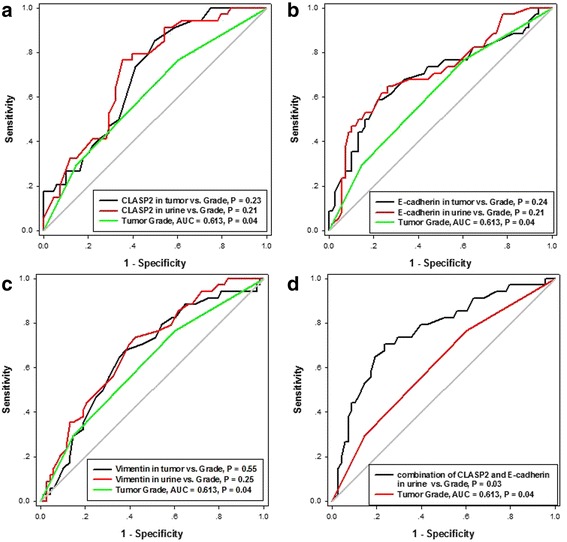
ROC curves used to compare the diagnostic potential of tumor grade and those of CLASP2 (**a**), E-cadherin (**b**) and Vimentin (**c**) mRNA levels in tumor and urine. The greater potential was shown in the combination of CLASP2 and E-cadherin mRNA in urine (**d**)

To evaluate the prognostic potential, ROC curves were generated for tumor grade, CLASP2, E-cadherin and Vimentin mRNA levels in tumor and urine (Fig. 4 and Table 3). Tumor grade were generally thought to be a strong predictive factor for poor prognosis in cancers. Area under curve (AUC) of ROC of tumor grade was compared with those of CLASP2, E-cadherin and Vimentin mRNA levels in tumor and urine. However, the results showed that mRNA levels of individual CLASP2 or other two EMT-related markers in tumor and urine could not discriminate better than grade. (*P* >0.05, Fig. 4 and Table 3). Table 3 indicated that the prognostic potentials of mRNA levels of CLASP2 and E-cadherin in urine were slightly greater than the others. And because urine samples were easily attained, the combined model (CLASP2 + E-cadherin mRNA in urine) was evaluated. The results showed that the combination could better discriminate the patients with or without progression compared with tumor grade (*P* = 0.03) (Fig. 4d).

**Table 3:** ROC^a^ curve analysis of CLASP2 and EMT markers for the discrimination of progression within 2 years

	AUC^b^	95% CI^c^	*P*
Tumor grade	0.613	0.512–0.708	0.02
CLASP2 mRNA (in tumor)	0.702	0.603–0.788	<0.01
CLASP2 mRNA (in urine)	0.716	0.619–0.801	<0.01
E-cadherin mRNA (in tumor)	0.692	0.593–0.780	<0.01
E-cadherin mRNA (in urine)	0.710	0.612–0.795	<0.01
Vimentin mRNA (in tumor)	0.657	0.544–0.768	0.01
Vimentin mRNA (in urine)	0.687	0.580–0.792	<0.01
Combination of CLASP2 and E-cadherin mRNA (in urine)	0.763	0.662–0.864	<0.01

^a^ROC: receiver operating characteristic

^b^AUC: area under the curve

^c^Confidence interval of AUC

## Discussion

In present study, we confirmed that manipulation of expression of CLASP2 could change the status of EMT in bladder cancer cell lines in vitro. Moreover, we investigated the mRNA levels of CLASP2 and EMT-related markers in both TURBT tumor samples and the corresponding patients’ urine. And we found out that the combination of mRNA levels of CLASP2 and E-cadherin in urine could be used as prognostic tools to predict the progression within 2 years after TURBT for treating patients with non-MIBC.

The significance of CLASP2 in EMT is not explicit, especially in malignant disease. CLASP2 is closely associated with the function of microtubules which regulate invasive protrusions, termed podosomes and their cancer counterpart’s invadopodia [21, 22]. Podosome-dependent extracellular matrix remodeling was implicated in cell migration and invasion during morphogenesis in several researches [23, 24]. CLASP is necessary for podosome formation. If the CLASP2 is depleted by siRNA, podosome numbers would be dramatically reduced as compared with those of cells treated with non-targeted control siRNA [8]. Previous results showed CLASP2 was enriched at the cortex of basal progenitor keratinocytes. Reductions in the levels of CLASP2 decreased the localization of the other protein to cell-cell contacts and altered adherens junctions dynamics and stability [6]. In our study, ectopic introduction of CLASP2 resulted in increased growth and aggressive phenotype of bladder cancer cells.

Although we did not study the changes of podosome or invadopoda in present study, we confirmed the involvement of CLASP2 in the cellular motility. After the expression of CLASP2 was up- or down-regulated by cDNA or shRNA, EMT-related markers including E-cadherin and Vimentin changed accordingly. Moreover, wound healing assay indicated that motile potential could be boosted greatly due to the overexpression of CLASP2. Cell migration is essential for development, tissue remodeling and wound healing, and requires coordination of intracellular signaling and cytoskeleton dynamics to generate traction forces. CLASP can promote the stability of peripheral microtubules [25].

However, the study of Nakaya et al. [11] seemed to be inconsistent with ours. They reported that CLASP2 is involved in gastrulation, one of the best-known examples of EMT. The function of CLASP2 was to maintain basement membrane integrity through their microtubules-binding ability during EMT under this circumstance [11]. Knockdown of CLASP leaded to premature basement membrane breakdown in lateral epiblast cells and initiated gastrulation EMT. We thought the reason of different roles of CLASP2 reported in two studies might be due to the different cell models (chicken epiblast cells vs. bladder cancer cells). And we hypothesized that CLASP2 might play varied roles during the diverse phases of EMT. During the early EMT, expression of CLASP2 might need to decrease so the cells could detach from the basement membrane. Then the expression would go up with the motile and invasive potential getting greater. These theories are worthy of further investigation.

A number of studies have proved the E-cadherin down-regulation at the transcriptional and protein level could be used to predict progression and poor prognosis for the patients with bladder cancer [26–28], which were consistent with our study. In univariable and multivariable analysis, low expressions of E-cadherin in tumor and urine cells were risk factors for progression within 2 years. However, the significance of soluble E-cadherin in urine differed from the expression in urine cytology. Shariat et al. [29] utilized ELISA assay to study the soluble E-cadherin in the urine of patients with bladder cancer. The results suggested that higher soluble E-cadherin was associated with an increased risk of bladder cancer and could be used as a tool to detect the recurrence. But the significance of soluble E-cadherin in bladder cancer progression was not included in their study. The correlation of E-cadherin between in tumor, urine cytology and urine itself needs more clarification.

No significance of CLASP2 in bladder cancer or urine has been reported. Therefore, we could not compare our findings with others concerning CLASP2. In our study, univariable and multivariable analysis suggested that high expressions of CLASP2 in tumor and urine cells were risk factors for progression within 2 years. These results suggest that CLASP2 is crucially implicated in the carcinogenesis and invasion of BC.

Few studies about the roles of Vimentin in bladder cancer had been published. The role of Vimentin in predicting progression of bladder cancer is obscure. Zhao et al. [30] used immunohistochemistry to test the expression of Vimentin in bladder cancer. They concluded that Vimentin is potential independent indicators in predicting bladder cancer progression and survival. But interestingly, Ohsaki et al. [31] found out that all the urothelial carcinoma cells in voided urine of their study were negative for Vimentin. The results from this study might not represent this particular urine cytology feature of bladder cancer because they only included low-grade bladder tumor. In our study, expression of Vimentin, as a mesenchymal marker changed when CLASP2 was up- or down-regulated in vitro experiments. But the univariable and multivariable analysis did not suggest any significance of Vimentin in predicting progression.

Since the results of logistic regression indicated CLASP2 and E-cadherin mRNA levels in tumor and urine were risk factors for progression of bladder cancer, the values of these biomarkers in discriminating the patients with 2-years progression from those without were further evaluated by ROC analysis. None of mRNA levels of individual CLASP2 or the EMT-related markers in tumor and urine showed better discriminating value than tumor grade (Fig. 3). But the combination of CLASP2 and E-cadherin mRNA levels in urine provided the significant discrimination between patients with and without 2-years progression compared with the grade (*P* = 0.03). To our knowledge, no other studies focusing on predicting early (2 years) progression after TURBT had been reported.

This study had some limitations. First, we confirmed the relationship between CLASP2 and EMT in bladder cancers, but the results were descriptive. The deeper molecular mechanisms need much more works and would be very interesting. Second, number of cases was relatively small. A larger sample size would ensure greater degree of accuracy in conclusions.

## Conclusion

In conclusion, CLASP2 is involved in the EMT and progression of bladder urothelial cancer. Simultaneous urine-based detection of CLASP2 and E-cadherin mRNA can efficiently discriminate patients with or without 2-years progression after TURBT.

## Abbreviations

BC: Bladder cancer
CLASP2: Cytoplasmic linker-associated protein 2
CLIP: Cytoplasmic linker protein
EMT: Epithelial-mesenchymal transition
non-MIBC: Non-muscle-invasive bladder cancer
TURBT: Transurethral resection of the bladder tumor
